# Supplementary material for: Are carbon emissions trading and green financial instruments synergistic? -Comprehensive quantitative research based on content analysis
Source: PLoS One. 2024 Mar 7;19(3):e0298601. doi: 10.1371/journal.pone.0298601 (PMC10919601; doi:10.1371/journal.pone.0298601)
Supplement: S1 Data — (ZIP) [file pone.0298601.s001.zip › Data/地级市Z_F工作报告.zip!/╔╜╢1⁄2/╤╠╠¿╩╨/└┤╫╘╣1⁄2╓┌║┼í╛┬φ┐╦╩2╛▌═°í┐_2004.docx]

各位代表：

现在，我代表市人民政府，向大会作政府工作报告，请予审议，并请各位政协委员和其他列席人员提出意见。

一、2003年政府工作的回顾

2003年是极不平凡的一年。一年来，全市人民坚持以“三个代表”重要思想和党的十六大精神为指导，在省委、省政府和市委的正确领导下，按照“三个明显、两个高于、一个跨越”的要求，解放思想，干事创业，齐心协力，扎实苦干，较好地完成了十四届人大一次会议确定的各项任务。全市国内生产总值初步核算为1316亿元，比上年增长17.4%，比全省平均增幅高4个百分点，在14个首批沿海开放城市中的排名由第8位前移至第7位。地方财政收入完成55.17亿元，增长26.7%。

——积极推进经济结构战略性调整，经济运行质量显著提高。坚持以加快发展为主题，大力调整产业结构、产品结构和所有制结构，全市经济呈现出速度加快、质量提高、后劲增强的良好态势。进一步优化农业结构，不断提高产业化、市场化、国际化水平，优势产业带和优质农产品生产基地建设成效明显。全市林牧渔业产值占农业总产值的比重达到52.1%。坚持工业立市、工业强市，加快培植主导产业、骨干企业和拳头产品，实现了工业经济速度效益的同步快速增长。规模以上工业完成销售收入1772.3亿元，实现利税189.1亿元、利润123.6亿元，分别增长47.8%、65.7%和80.9%。制定了建设北部沿海经济产业带总体规划和机械制造、电子信息、食品加工、黄金四大支柱产业发展规划，并付诸实施，四大支柱产业销售收入和利税分别占规模以上工业的57%和59%。高新技术产业发展迅速，实现产值406.3亿元，增长48.7%。服务业积极克服非典影响，继续稳定增长。全市社会消费品零售总额达到366.1亿元，增长16%，旅游业总收入达到71.6亿元，金融机构各项存贷款分别比年初增加168.8亿元和164.2亿元。大力发展非公有制经济，全市民营经济实现增加值736亿元，占国内生产总值的比重由上年的54.3%提高到56.1%。

——众志成城抗击非典，取得阶段性重大胜利。去年上半年，面对突如其来的非典疫情，全市上下万众一心，众志成城，坚持一手抓非典防治不放松，一手抓加快发展不动摇，自上而下建立了强有力的指挥机构，健全了严密的群防群控网络和疫情报告网络，坚持严防、严控、严查、严备，认真落实各项防治措施，有效杜绝了疫病输入。与此同时，深入开展爱国卫生运动，大力加强公共卫生体系建设，建立了公共卫生管理和应对突发事件的长效机制，依法规范了卫生医疗市场和药品市场秩序。

——大力实施经济国际化战略，对外经贸取得新的突破。坚持以对外开放统揽经济工作全局，发动全社会力量开展招商引资，开拓国际市场，掀起了对外开放的新高潮。全市合同利用外资40.1亿美元，实际利用外资20.5亿美元，分别增长79.3%和70.1%。外贸进出口总额完成58.9亿美元，增长31.6%，其中出口33.3亿美元，增长29.6%。外贸依存度由上年的33.3%提高到37.1%。积极支持各类企业“走出去”，劳务输出和工程承包均有较大增长。引进内资工作取得重大进展，全年共引进国内资金128.5亿元。园区建设水平不断提高，出口加工区B区经国务院批准正式设立，12个省级以上园区实际利用外资和外贸出口分别占全市总额的34.8%和42.7%。投资环境进一步优化，亲商、富商、安商的氛围更加浓厚。第五届国际果蔬博览会及境内外一系列重大经贸活动取得丰硕成果。

——纵深推进各项改革，市场经济运行机制日臻完善。以建立完善的社会主义市场经济体制为目标，进一步改革宏观管理体制和微观运行机制。积极推进国有经济结构和布局的战略性调整，国有资本有序进退、流转顺畅的营运机制初步形成。坚持以产权制度改革为突破口，全面推进企业改革，一批国有大企业成功改制，一批劣势企业顺利退出市场。全市实行产权制度改革的企业达到81.3%。通过企业上市、增发新股和配股，当年共从资本市场直接融资7.4亿元。深化农村税费改革，全面停征乡村公益事业金，5个市、区取消了农业特产税。大力推进了城市规划建设管理体制、市政公用事业管理体制和港航管理体制等方面的改革。

——进一步加快城市建设步伐，城乡面貌发生新的变化。按照“年年有变化、三年见成效、五年大变样”的要求，不断提高城市的规划、建设、管理和经营水平。大规模增加城建投入，集中用于重大基础设施和市政公用设施建设，用于环境建设和综合整治工程，全年市区完成城建投资45亿元。新建、改造了210多公里的城市主次干道，完成了连城路、山海路、塔山隧道、滨海东路等一批重点道路工程，“拓展东西两翼、贯通南北山海”的城市建设框架进一步拉开。滨海广场、机场改造、体育公园、养马岛跨海大桥等重点工程进展顺利。国道206高速公路烟台段全线贯通，全市高速公路通车里程达368.8公里，实现了市市通有高速公路。进一步改进城市管理方式，提高城市管理水平，老城区和南大街综合整治工作成效明显，荣获“山东人居环境范例奖”和“国家人居环境范例奖”。注重规划的先导作用，全面实施“阳光规划”，城市总体规划编制大纲已全面完成，控制性详细规划编制加速推进。认真清理整顿土地市场，推进土地资源的市场化配置，对所有经营性土地一律实行“招拍挂”，全市实现土地纯收益21亿元。县级市和小城镇建设步伐加快。全市城市化水平达到49%，比上年提高1.1个百分点。

——持续加大有效投入，经济发展后劲明显增强。注重发挥投资对经济增长的拉动作用，拓宽资金融通渠道，加强重点项目调度，不断培植新的经济增长点。全社会固定资产投资完成737.2亿元，其中技改投资165.2亿元，分别增长75.8%和46.7%。总投资千万元以上开复工项目989个，完成年度投资439亿元，527个项目竣工投产。上海通用东岳汽车项目批量生产，当年生产轿车3.8万辆。南山铝材、万华16万吨MDI、玲珑橡胶工业园等一批大项目开工建设。烟大铁路轮渡、黄烟铁路和天然气城市供气工程进度加快。海阳核电、海上油气田陆上基地、胶东供水等重大项目顺利推进。

——高度重视各项社会事业，精神文明和民主法制建设取得新的进展。深入实施科教兴烟战略，推进科技创新和教育事业健康发展。全市52个科技项目列入国家科技计划，其中10项列入“十五”攻关计划和“863”计划。“以县为主”的教育管理体制全面实行，义务教育均衡发展，优质高中教育资源进一步扩大，高等教育规模快速膨胀。深入开展建设体育强市活动，群众体育普遍开展，竞技体育水平显著提高，在全国第五届城市运动会和全省农民运动会上，均取得优异成绩。积极开展“诚信烟台”建设活动，社会信用体系不断完善。繁荣发展文化事业，一批优秀文艺作品在全国、全省获奖。深入开展双拥共建活动，烟台市、长岛县被评为全国双拥模范城（县）。环境保护、人口与计划生育工作取得新进展。国防动员、民兵预备役和人防工作进一步加强。高度重视安全生产和社会治安综合治理工作，有效维护了社会稳定。基层民主继续扩大，基层自治组织不断健全。广播电视、新闻出版、气象地震、史志档案、外事侨务、民族宗教、妇女儿童、残疾人等各项社会事业都有新的发展。

——真心实意为群众办实事，人民生活水平进一步提高。全年城镇居民人均可支配收入达到9785元，农民人均纯收入达到4120元，分别增长10.3%和7.7%。年初向市民承诺的十件实事全部完成。高度重视就业再就业工作，进一步强化社会保障体系建设，全年城镇新增就业10.2万人，低保金社会化发放率达到100%。积极开展新型农村合作医疗试点，认真解决拖欠职工和农民工工资问题，探索建立失地农民基本生活保障制度。狠抓信访和市长公开电话工作，有效解决了一大批人民群众关心的热点、难点问题。立足于改善居民居住条件，对市区18个社区、25个居民小区进行了综合整治。加大路网管线和绿地建设力度，市区新增供热面积100万平方米，新增公共绿地330万平方米，更新城市公交大巴262辆。对生活特困群众和弱势群体进行重点帮扶，新建经济适用房1000套、廉租房100套，对1000户残疾人进行脱贫帮扶，14多万人次家庭贫困中小学生得到就学资助，建设完善了20处老年公寓和80处老年活动场所。

——努力创建“阳光政府”和“责任政府”，政府自身建设进一步加强。各级政府按照市场经济要求，转变职能，简政放权，不断提高执政能力。完善决策机制，规范工作程序，大力实施“阳光政务”工程，开通政府门户网站，创办政府公报，提高了政府工作的透明度。加强行政效能监察，深化行政审批制度改革，再次取消、合并、下放和变更审批事项105项，实现了政府工作提速增效。深入开展廉政建设和反腐败斗争，部门和行业风气继续好转。

回顾过去的一年，我们深深体会到，面对复杂局面和诸多矛盾，要战胜困难，不断夺取新的胜利，必须始终不渝地坚持解放思想、与时俱进，勇于突破自我、高点定位，以思想的大解放推动经济社会的快速发展；必须牢牢把握加快发展这个执政兴国的第一要务，以结构调整为主线，以改革开放和科技进步为动力，聚精会神搞建设，一心一意谋发展；必须立足烟台的特点和优势，进一步明确工作思路，抓好事关长远的重大战略性问题，集中用力、重点突破；必须大力弘扬求真务实、狠抓落实的工作作风，对重大决策和部署一抓到底，确保落实；必须认真贯彻立党为公、执政为民的本质要求，把维护和实现人民群众的根本利益作为政府工作的出发点和落脚点，千方百计为群众排忧解难，把方方面面干事创业的积极性保护好、引导好、发挥好。实践证明，只要我们牢记党的宗旨，保持高昂的斗志、科学的精神和扎实的作风，一切为了群众，一切依靠群众，就能战胜任何困难和挑战，就能应对各种复杂局面，烟台跨越发展的目标就一定能够实现！

各位代表，过去一年的成绩来之不易。这是在省委、省政府和市委正确领导下，全市人民开拓奋进和共同努力的结果，是社会各界和海内外友好人士热情参与和积极支持的结果。在此，我代表市政府，向全市广大工人、农民、知识分子以及各阶层的建设者，向各位人大代表和政协委员，向各民主党派、各人民团体、社会各界人士，向人民解放军、武警驻烟部队全体指战员和公安干警，向中央、省属驻烟单位，向所有关心支持烟台建设的港澳同胞、台湾同胞、海外侨胞和国际友人，致以崇高的敬意和衷心的感谢！

各位代表，我们还要清醒地看到，在前进道路上还存在一些矛盾和问题，主要表现为：经济结构不够优化，第三产业相对滞后，民营经济不够活跃，城乡二元结构的矛盾仍比较突出。经济发展中的体制性障碍仍然存在，有些领域的改革措施还不到位。“三农”问题仍很突出，农民增收困难较大。社会保障体系不够完善，就业再就业压力较大。经济外向度低，出口产品结构不合理，开放环境需要进一步改善。社会事业发展与经济发展不相适应。政府职能转变还不到位，少数部门和工作人员服务意识不强、效率不高、作风不实等问题仍然存在。这些都需要我们在今后的工作中采取有效措施，认真加以解决。

二、2004年政府工作的安排

2004年是深入贯彻十六大和十六届三中全会精神，解放思想、跨越发展的重要一年，也是实现“十五”计划的关键一年。国家宏观政策保持稳定，国际经济增长回升，我市加快发展的基础越来越好，广大干部群众的发展热情十分高涨。尽管我们还将面临更直接、更激烈的竞争和挑战，尽管我们还会遇到更严峻、更复杂的困难和问题，但我们完全有信心、有能力夺取经济社会发展的新胜利。今年的政府工作，要坚持以邓小平理论和“三个代表”重要思想为指导，全面贯彻党的十六大和十六届三中全会精神，牢固树立全面、协调、可持续的科学发展观，按照“五个统筹”、“五个坚持”的要求，进一步解放思想，狠抓结构调整，扩大对外开放，深化各项改革，加快城市化进程，认真解决“三农”问题和就业问题，转变经济增长方式，提高经济运行质量，改善城乡人民生活，促进国民经济持续快速协调健康发展和社会全面进步。

国民经济和社会发展的主要预期目标是：国内生产总值增长14%；地方财政收入增长19%；全社会固定资产投资增长22%；社会消费品零售总额增长16%；城镇居民人均可支配收入增长9%，农民人均纯收入增长5%；城镇登记失业率控制在3.7%以内。实际工作中要加倍努力，力争发展得更快更好。

围绕实现上述目标，突出抓好以下工作重点：

（一）全力推进对外开放，更加积极地参与国际经济技术合作与竞争。继续把对外开放放在突出位置来抓，不断提高经济外向度和国际竞争力。一是，大力提高利用外资水平。紧紧围绕培育壮大四大支柱产业，特别是建设国际汽车及零部件基地和打造“中国食品名城”，集中引进一批产业关联度高、辐射带动力强的大项目，提高招商引资的针对性。加大市政公用设施和基础设施利用外资力度，扩大商贸、旅游等领域的对外开放，引导外资向采购、服务、营销和研发等方面延伸，使我市不仅成为重要的制造业基地，而且成为重要的采购基地、服务基地和研发基地。创新招商方式和招商机制，重点开展专业招商、产业招商和代理招商，鼓励现有外资企业增资扩股。特别要下大气力推动大企业与世界500强的合作，使大企业率先进入跨国公司的生产和营销体系。办好APEC电子商务博览会、第六届国际果蔬博览会等重大经贸活动。进一步加快园区建设，增强载体功能，培育产业特色，提高对外吸引力和项目集聚度。启动出口加工区B区的开发建设，实现开发区、出口加工区优势互补。年内，全市实际利用外资增长27%。二是，努力扩大外贸出口规模。按照国家出口退税改革的新要求，落实出口退税的地方资金和各项鼓励政策。调整优化出口结构，提高传统出口产品的竞争力，提高机电产品和高新技术产品出口比重。大力发展加工贸易，促进“落地生根”和转型升级。年内，全市外贸出口增长15%。三是，积极实施“走出去”战略。完善对外投资服务体系，鼓励有比较优势的企业走出国门投资办厂、承揽工程、开展劳务合作，发展带料境外加工，建立资源开发基地和产品营销网络。四是，千方百计引进内资。坚持“烟台以外都是外”，主动承接先进地区特别是珠江三角洲和长江三角洲的产业转移。年内，力争引进内资165亿元。五是，进一步优化投资环境。不断提升各类服务的标准、质量和水平，降低商务运行成本。加强口岸建设，完善“大通关”机制。重视外商投诉处理，提高对外商的亲和力和吸引力。

（二）全面深化市场取向的各项改革，推进机制创新和体制创新。按照十六届三中全会《决定》的要求，统筹兼顾，规范运作，扎实推进重点领域的改革。一是，推进国有企业改革。以建立现代产权制度为核心，推进投资主体多元化，发展混合所有制经济，使股份制成为公有制的主要实现形式。年内，争取完成65户市级企业的产权制度改革，重点推进张裕、万华、冰轮等企业部分国有产权的对外转让和整体改制。加大政策支持力度，加快国有劣势企业退出市场步伐。抓好上市公司再融资，培育上市后备企业，争取有新的企业上市。二是，深化国有资产管理体制改革。推进政府公共管理职能和国有资产出资人职能分开，建立出资人权利、义务、责任相统一，管资产和管人、管事相结合的国有资产监管体制。发展和规范产权交易市场，健全国有资本有进有退、合理流动的营运机制。三是，完善就业和社会保障体系。坚持劳动者自主择业、市场调节就业和政府促进就业的方针，加大再就业援助力度，规范企业用工行为，加快建立政府统一领导、部门各负其责、社会广泛参与的社区就业管理体制。年内，新增城镇就业8万人。完善企业职工基本养老保险制度，全面实行生育保险，进一步健全医疗保险和工伤保险。强化社会保险基金征缴，完善城乡居民最低生活保障和社会救助制度，健全农村社会养老保险制度。四是，深化财税体制改革。优化财政支出结构，健全公共财政体制，进一步深化部门预算、国库集中收付、政府采购和收支两条线管理改革。理顺分税制财政体制，进一步规范市对下的财政分配关系。五是，整顿规范市场经济秩序。完善行政执法、行业自律、舆论监督、群众参与相结合的市场监管体系，健全产品质量监管机制，严厉打击制假售假、商业欺诈等违法行为。六是，推进社会事业改革。以激发活力为重点，加快事业单位综合配套改革，真正建立与市场经济体制相适应的管理体制、人事和分配制度。进一步落实好城市公用事业改革实施意见，加快城市供水、供热、供气等公用事业市场化进程。

（三）大力调整工业经济结构，加快建设制造业强市。以建设胶东半岛制造业基地为总抓手，加快调整产业结构、产品结构和企业组织结构，努力把制造业做大做强。一是，集中培植壮大支柱产业。继续膨胀发展机械制造、电子信息、食品加工和黄金四大支柱产业，努力使总量规模、技术水平和市场占有率实现大的突破。机械制造业，重点发展汽车、船舶、工程机械、数控机床及制冷设备，加快建设汽车及零部件工业园区。电子信息业，重点抓好IT产业园、软件园和信息产业城建设，大力发展通信终端设备、计算机及外围设备、电子元器件和新型电子材料，推进高清晰度荫罩、高速数据电缆等10个重点项目的开发建设。食品加工业，重点抓好果蔬、酿酒、水产、粮油、畜禽五大行业，努力创建“中国食品名城”。黄金产业，突出抓好精深加工，扩大黄金交易规模，把我市建成黄金生产、加工、交易的聚集地。年内，四大支柱产业实现增加值370亿元，占规模以上工业的比重达到58%。与此同时，加快培植纺织服装、生物医药和新材料等优势产业，不断扩大生产规模，增强市场竞争能力。二是，大力培植骨干企业。实施名牌名企战略，积极推进企业强强联合和资产重组，大力培植张裕、万华、冰轮、南山等传统骨干企业，加速膨胀东岳汽车、大宇重工、浪潮LG、泰信电子等新兴骨干企业，尽快培育一批具有较高知名度和较强竞争力的拳头产品，争创更多的国家驰名商标。三是，加快企业技术创新步伐。大力推进高新技术产业化和传统产业高新化，广泛运用高新技术和先进实用技术改造提升传统产业。抓好制造业信息化工程建设，以信息化带动工业化。年内，全市力争完成技改投资200亿元，增长21%，重点抓好氨纶和正海扩建等50个投资过亿元的重大技改项目；高新技术产业产值达到520亿元，增长28%。

（四）下大气力抓好“三农”工作，更好地统筹城乡经济社会发展。坚持把解决“三农”问题作为经济工作的重中之重，按照统筹城乡经济发展的要求，坚持“多予、少取、放活”，促进农民增收、农业发展、农村社会全面进步。一要纵深推进农业产业化经营，带动农业结构优化升级。继续把培植壮大龙头企业作为关键环节来抓，鼓励工商资本、民间资本和外资投向农产品加工业。大力兴办各类合作经济组织，提高农民的组织化程度。积极培育适销对路的特色种养业，特别是以食草为主的畜牧业和附加值高的海产品，提高农产品的优质化率和增值能力。优化农业区域布局，建设农产品优势产区和产业带。二要完善社会化服务体系，扩大农产品销售。继续强化农业产前、产中、产后服务，加快农业新技术、新成果的研发推广，完善农业信息化服务体系。加强农业标准化建设，建立健全市县两级农产品质量检测体系，鼓励龙头企业争取国际质量体系认证，创建知名品牌。继续搞好以果品为主的农产品促销活动，促进农产品进城出境。年内，全市农产品出口创汇达到13亿美元。三要加快农村劳动力转移，拓宽农民增收渠道。大力发展农村二三产业，加强农村劳动力素质技能培训，加快建立农村劳动力就业服务体系，建设城乡统一的劳动力市场，改善进城务工农民的就业环境。继续实施“双五○”工程，年内由第一产业转移劳动力20万人，由农民转市民20万人。四要增加农业投入，夯实农业发展基础。建立支农资金稳定增长机制，加强农机、水利、道路等基础设施建设，加快发展农村社会公共事业，改善农民生产生活条件。年内重点抓好小流域综合治理、村村通油路和农村人畜饮水解困工程。五要落实党的农村政策，切实保障农民利益。按照国家和省统一部署，全面取消农业特产税，改征农业税，并降低农业税税率1个百分点；调整粮食补贴政策，把对粮食流通环节的间接补贴改为对种粮农民的直接补贴，保护粮食安全。进一步稳定土地承包关系，规范土地承包经营权流转办法。严格土地保护政策，完善土地征用补偿办法，关心和解决失地农民的生产生活问题。推进村务公开，搞好农村集体财务与资产管理。认真开展专项治理，严肃查处损害农民利益的案件，确保农民负担明显减轻不反弹。

（五）大力发展现代服务业，促进城乡经济繁荣。加快改组改造传统服务业，积极发展现代服务业，不断提高服务业整体水平和第三产业在国民经济中的比重。一是，突出服务业发展重点。以建设滨海生态旅游城市为目标，加快旅游基础设施建设，深度开发和整合旅游资源，推进旅游区(点)所有权和经营权分离，打造“黄金海岸、人间仙境”的城市品牌，做大做活旅游业。积极推进流通现代化，发展连锁经营、物流配送、现代批发市场等新型流通业态，集中培育大型流通企业、大型批发交易市场，引进一批大型现代物流集团，争取和建设保税物流园区。积极引进和发展国内外特色餐饮，提升餐饮业的风味特色和文化品位。引导商贸、餐饮、娱乐企业延长营业时间，繁荣发展夜间经济。进一步拓展金融保险服务领域，提高金融服务科技含量，完善保险保障功能，加强金融监管，防范金融风险。加快发展会计、法律、咨询、评估等中介服务业，积极培育人才、物资、土地、证券等要素市场。二是，抓好服务业载体建设。搞好港口规划布局，加强资源整合，以港口为依托，扩大集装箱规模，发展临港产业，完善机场、铁路服务功能，建立现代综合运输体系和专业物流服务网络，增强人流、物流承载能力。全面实施《烟台市市区商业网点发展规划》，抓好市、区两级商业中心建设，集中建设好十个重点服务设施项目。三是，努力扩大消费需求。通过发展经济和完善社会保障体系，规范分配秩序，理顺分配关系，努力提高中、低收入者的收入水平，提高城乡居民消费能力。扩大消费信贷，引导居民转变消费观念，加快培育住房、汽车、旅游、文化、健身等新的消费热点。四是，加大政策扶持力度。创新体制机制，切实降低门槛，消除价格歧视，创造公平竞争的市场环境。落实好国家有关税收优惠政策，落实政府服务业发展引导资金，广泛吸引国内外资金发展服务业。年内，全市服务业增加值力争完成555亿元，占国内生产总值的比重比上年提高1个百分点以上。

（六）继续加大有效投入，不断优化投资结构。加大投入的重点要继续放在重大项目建设上，突出抓好1359个投资1000万元以上的重点项目调度，建立项目建设绿色通道，提高项目开工率，确保投资到位率。继续推进海阳核电、IGCC、渤海天然气供气、海水淡化、黄烟铁路、烟大铁路轮渡、国道206线烟蓬段、胶东引黄调水工程和八角港区等一批重大项目建设，争取前期工作项目取得突破性进展，在建项目进度明显加快。精心筛选一批投资规模大、技术水平高、带动能力强的后备项目，搞好储备项目的论证和实施，为经济持续增长储备力量。调整优化投资结构，引导资金重点投向现代制造业、高新技术产业、现代服务业和传统产业的技术改造，投向重点园区、骨干企业和名牌产品，投向基础设施、环境保护和社会事业。充分发挥市场的主导作用、政府的引导作用和企业的主体作用，拓宽资金融通渠道，争取政策性投入，扩大信贷投入，激活社会投入，吸引跨国公司战略投入。年内，确保完成全社会固定资产投资900亿元。

（七）创新发展县域经济和民营经济，增强区域经济活力。县域经济是我市最大的经济板块，要作为增强全市综合实力、增加农民收入、统筹城乡发展的战略举措来抓。一是，实施积极的非均衡发展战略。坚持扶弱促强，鼓励经济强县率先发展，中等县快速崛起，困难县跨越式赶超。集中力量抓好北部沿海经济产业带建设，加强县域间的产业分工与合作，形成产业联盟，共同做大做强优势产业。加快重要基础设施的共建共享，实现信息、资本、物流等要素的快速流动，并通过建立健全协调机制，尽快实现一体化发展。二是，大力发展特色经济。加强分类指导，引导各县市区立足区位、资源和产业等方面的比较优势，选准发展方向，把潜在的资源优势转化为现实的经济优势，大力培植有竞争力的主导产业，实现优势互补、错位竞争、共同发展。三是，继续推进简政放权。建立健全权责统一的管理体制和运行机制，加强和完善县级政府的经济、社会管理权限，赋予县级政府更大的发展自主权。

民营经济蕴藏着巨大的发展潜力，要摆到更加突出的位置，千方百计创造条件，促其膨胀规模、提升档次。一是，进一步拓展发展空间，扩大民营经济总量。消除体制性、机制性障碍，放宽市场准入，凡允许外资进入的领域，凡法律法规未禁入的领域，都要允许民营资本进入。二是，鼓励民企做大做强，提升整体素质。坚持大中小并举，重点扶持一批主业突出、管理先进、核心竞争力强的优势企业，引导中小民营企业进区入园，形成区域性产业集群。鼓励民营经济参与国有企业改制重组，推进民营企业制度、技术和管理创新，促其向科技型、外向型、集团化方向发展。三是，强化政策扶持，优化发展环境。全面落实扶持民营经济发展的政策措施，在投融资、税收、土地使用和对外贸易等方面，与其他企业享受同等待遇。尽快健全完善民营企业信用担保、诚信评估、人才培训、法律服务体系，切实解决民营企业融资困难、信息不灵、人才缺乏等问题，努力激发全民创业热情。

（八）提升城市整体形象，加快城市化进程。以建设半岛城市群为契机，努力推动城市向高起点规划、高标准建设、高效能管理迈进，争创“国家人居环境奖”，全面建设生态市。一是，强化规划的先导作用。按照以人为本和经济与社会、人与自然协调发展的要求，搞好规划编制，尽快完成城市总体规划调整，细化建成区的控制性详细规划，年内控规覆盖率达到100%。完善经济园区、重要风景带、历史文化街区等专业规划。理顺规划管理体制，依法加强规划监管，增强规划的控制力。二是，继续加大建设力度。在进一步拉开城市发展框架的同时，着力提高城市内涵。建设重点向标志性、公益性和旧城改造倾斜，向山岛开发和港口建设倾斜，抓好滨海广场、中心街区改造、体育公园等续建工程，抓好火车站、红旗路东拓、只楚路整治、二马路拓宽、胜利路延伸、区河整治等新建工程。切实加强和改进投资项目管理，严格实行项目法人制、工程招投标制、工程监理制和合同管理制，保证工程质量，提高投资效益。三是，创新城市管理。尽快落实新的管理体制，加快市、区两级职能到位，形成完善的“两级政府、三级管理、四级网络”的管理体制。市区要加强市容市貌、市政公用设施维护，搞好集贸市场、城乡结合部、居民小区等重点区域的综合整治，不断提高整个城市的洁绿亮美水平。完善环境基础设施，强化环境保护，统筹城乡绿化美化，建设“生态城市、绿色烟台”。四是，加快县级城市和小城镇建设。鼓励有基础的县级市向中等城市迈进，规划发展一批各具特色的小城镇。有条件的地方要同步规划城乡基础设施建设，使现代交通、通讯、供电、供排水、信息网络逐步向农村延伸覆盖，提高农村基础设施配套水平。五是，搞活城市经营。建立权责明确、制约有效、科学规范的经营城市机制，加强城市资源综合开发。搞好土地储备，加大“招拍挂”实施力度，最大限度获取土地收益，年内全市力争实现土地纯收益25亿元。搞好城市无形资产经营，推动市政公用设施对外出让，有效盘活存量资产。

（九）全面推进科教兴市，统筹发展各项社会事业。坚持以教育为基础，以科技为关键，以人才为根本，不断完善适应现代化建设需要的区域性现代教育体系、科技创新体系和人才支撑体系，推进各项社会事业全面繁荣。一是，加快发展教育事业，建设学习型社会。高质量、高水平、均衡化发展义务教育，大力发展学前教育、职业教育和成人教育，加快发展高等教育。完善和规范以政府投入为主、多渠道筹措经费的教育投入体制，鼓励社会力量办学，形成公办学校和民办学校共同发展的格局。二是，积极推进科技进步，增强科技创新实力。改革科技管理体制，继续加大科技投入，加快科技创新体系建设。确立企业技术创新和科技投入的主体地位，加大对科研人员和科技成果的奖励力度。搞好产学研联合，进一步密切同中国科学院、中国工程院以及清华、北大等院校的合作，加快高新技术项目的引进和成果转化。抓好留学人员创业园区、中俄基地和三校科技园建设，构筑高新技术产业发展的良好平台。大力发展电子商务、电子政务，积极推进国产软件试点和“市民卡”应用。三是，创新人才工作机制，实施人才强市战略。强化人才是第一资源的观念，抓住培养、引进、使用人才三个环节，建立优秀人才脱颖而出和人尽其才、才尽其用的有效机制和良好环境。四是，大力发展先进文化，全面繁荣各项社会事业。加大文化投入，改善公益性文化设施，创作更多的文化精品。推进经营性文化的市场化步伐，培育一批文化名牌产品、一批文化企业集团。深化卫生体制改革，强化政府公共卫生管理职能，完善公共卫生设施。建设市疾病控制中心，改造市传染病医院。扩大农村新型合作医疗制度试点，加强与国内外知名医院的合作。深入开展科技、文化、卫生“三下乡”活动，促进农村社会事业加快发展。积极筹办第6届市运会，努力申办第21届省运会。加强居民小区体育健身设施建设，完善群众体育服务体系。加强人口与计划生育工作，稳定低生育水平，提高出生人口素质。高度重视史志档案、民族宗教、妇女儿童、残疾人和老龄工作，大力发展新闻出版、广播电视、气象地震等各项社会事业。

（十）推进精神文明和民主法制建设，保持政治稳定、社会安定。大力培育和弘扬民族精神，不断提高全民的思想道德素质和科学文化素质，为改革发展提供强大的精神动力和智力支撑。深入开展群众性精神文明创建活动，宣传普及现代科学知识，倡导健康文明的生活方式。做好群众来信来访工作，健全落实矛盾纠纷排查调处工作机制，积极妥善处理人民内部矛盾特别是涉及群众切身利益的矛盾，最大限度地减少民转刑案件和群众上访事件。加强国家安全工作，深化严打整治斗争，抓好社会治安防控体系建设，努力创建“全国最安全城市”。抓好安全生产，努力防止各类事故的发生。加强信用道德建设，深入开展“诚信烟台”活动。深化国防教育，加强民兵预备役、人防和优抚安置工作，深入开展双拥共建活动，支持部队现代化建设。

三、切实加强政府自身建设

做好今年的政府工作，必须进一步加强自身建设，努力使各级政府素质更高、能力更强、作风更实。

坚持解放思想、与时俱进，努力开拓政府工作新境界。各级政府公务员特别是领导干部要深入持久地学习实践“三个代表”重要思想，深刻领会十六大和十六届三中全会精神，与党中央在思想上、政治上保持高度一致。不断学习新知识，研究新情况，解决新问题，提高科学判断形势的能力、驾驭市场经济的能力、应对复杂局面的能力和依法行政的能力。正确把握形势，客观分析自我，在全国、全省发展大格局中确立工作定位，始终保持永不自满、永不懈怠的进取动力。牢固树立全面科学的发展观，自觉用“五个统筹”、“五个坚持”的要求指导政府工作，更加重视结构调整、效益提高、产业升级，更加重视政治、经济、文化的协调发展，更加重视人与自然的和谐，促进人民生活不断改善、社会文明程度日益提高。

坚持立党为公、执政为民，扎扎实实为群众办实事、办好事。人民的利益至高无上。要紧紧抓住人民群众最现实、最关心、最直接的问题，为群众诚心诚意办实事，尽心竭力解难事，坚持不懈做好事，使政府各项工作真正体现群众意愿、符合群众利益、满足群众需求，不断使群众从经济社会发展中得到更多实惠。在城市，要重视解决好困难家庭住房、子女上学、就医看病等基本生活需求问题，解决好下岗职工特别是“双下岗”家庭的再就业和生活问题。加大市直部门包帮生活特困户和就业困难户的力度，继续做好“两个确保”和“一个低保”的衔接工作。在农村，要重视解决好优抚对象、特困家庭、鳏寡孤独、受灾群众的生产生活问题，支持困难地方农村中小学危房改造和乡镇卫生院建设。要逐步探索和建立帮扶工作的制度和机制，使扶贫济困工作步入规范化、长效化轨道。继续办好一批为民服务的实事，切实解决部分群众的实际困难。

坚持依法行政、从严治政，努力建设公正透明、廉洁高效的人民政府。强化政府的经济调节、市场监管、社会管理和公共服务四个方面的主要职能，不断完善与社会主义市场经济体制相适应的行政管理体制。认真学习贯彻《行政许可法》，推进行政审批制度改革。全面提高政务公开水平，落实人民群众的知情权、参与权、选择权和监督权，完善重大决策的规则程序，完善群众建议征集制度、重大事项社会公示制度、社会听证制度和专家咨询制度。进一步抓好政府机关工作人员特别是领导干部的廉洁自律，坚决查处各种违纪违法案件。坚持标本兼治、综合治理，注重思想道德教育，完善监督制约机制，铲除不正之风和腐败现象滋生的土壤。加大行政监察力度，重点解决滥用行政执法权力、行政不作为、工作推诿扯皮等问题。建立健全决策目标、执行责任和考核监督“三个体系”，进一步营造干事创业的良好氛围。自觉接受市人大及其常委会的法律监督、工作监督和市政协的民主监督，自觉接受群众监督和舆论监督，高度重视人大代表建议和政协提案的办理工作，广开参政议政渠道。充分发挥各民主党派、工商联、人民团体以及社会各界的作用，凝聚社会一切积极因素，共同推进社会主义物质文明、政治文明和精神文明建设。

各位代表，我们已经踏上了跨越式发展的新征程。要在更高的起点上实现更大的发展，任务更加艰巨，必须付出更大努力。让我们更加紧密地团结在以胡锦涛同志为总书记的党中央周围，坚持以邓小平理论和“三个代表”重要思想为指导，全面深入贯彻十六大和十六届三中全会精神，在中共烟台市委的坚强领导下，解放思想，与时俱进，扎实工作，埋头苦干，努力夺取我市改革开放和现代化建设的新胜利。
